# Predictors of Treatment Outcome for Retreatment Pulmonary Tuberculosis Cases among Tribal People of an Eastern India District: A Prospective Cohort Study

**DOI:** 10.1155/2016/8608602

**Published:** 2016-08-30

**Authors:** Rajib Saha

**Affiliations:** Department of Community Medicine, Bankura Sammilani Medical College & Hospital, Bankura, West Bengal 722101, India

## Abstract

*Objective*. The study was conducted to assess the treatment outcome of different category retreatment cases with the aim of finding out the important predictors of unfavorable outcomes.* Methodology*. This hospital based prospective cohort study was conducted in three tuberculosis units (TUs) of west Midnapore (a district of Eastern India), covering mostly the tribal populated areas. Patients who were registered for Category II antituberculosis treatment between 1st quarter of 2013 (Jan to Mar) and 4th quarter of 2013 (Oct to Dec) were considered as our study cohort and they were followed up till December 2014. The study was started with 177 patients but ultimately ended with 165 patients.* Results*. Unfavorable outcome was observed among 24.8% patients. Among them mostly 51.2% were defaulter, 22% were failure case, and 26.8% died during treatment. Patients, who were minority by religion, were found 4 times more vulnerable for unfavorable outcome. Unfavorable outcome was found 7 times more common among retreatment TB cases who remain sputum positive after completion of initiation phase of Category II treatment.* Conclusion*. Programmatic approach should be specified to address the minority by religion population and to reduce the load of sputum positive cases after completion of initiation phase treatment by tracking them.

## 1. Introduction

Tuberculosis (TB) remains a major cause of morbidity and mortality worldwide, despite increased disease notification rate, spreading of antitubercular programmes. It is also a sociological, economical, and mental burden for a society. The World Health Organization (WHO) estimates that one-third of the population of the world is infected with* Mycobacterium tuberculosis*. Globally more than 9.8 million new cases of active TB are notified annually and 2-3 million deaths occur in every year [1, 2]. In 2011, 2.3 million new TB cases and 43000 retreatment pulmonary TB cases were notified in India [3].

Patients, who fail, default, or relapse after completion of standard first-line TB treatment and present for retreatment, are grouped together as Category II cases by the World Health Organization (WHO). In India where individual drug susceptibility testing (DST) settings are not universally accessible till now, there patients are often treated with a standard retreatment regimen of first-line agents (a regimen that adds a single drug to the standard initial TB treatment regimen) [4]. Retreatment case's outcomes often are found poor as MDR-TB, especially in patients with treatment failure or default cases [5].

Inappropriate implementation of the Revised National Tuberculosis Control Programme (RNTCP) causes precipitation of MDR-TB cases in the community. In this situation, India is not well equipped to prevent the propagation and dissemination of MDR-TB cases. So a new reemerging threat is slowly growing within the Indian population that may arise as a big challenge in future. MDR-TB is a man-made phenomenon; poor treatment, poor drugs, and poor adherence lead to the development of MDR-TB [6].

The tribal populations of Eastern India are likely to live in particular discrete hard to reach geographic areas with their common cultural and socioreligious beliefs which are quite different from the general population. The tribes are an underprivileged community and always remain away from the light of civilization and often with poor access to the health care system. These factors make a communication barrier with all health care facilities and make them more vulnerable to develop drug resistant TB.

Smear +ve and −ve previously treated pulmonary TB cases are suspected as MDR-TB case according to Programmatic Management of Drug Resistant TB (PMDT) guideline. But in India where drug resistance TB diagnosis facilities are not available widely till now, there unfavorable outcomes of retreatment TB cases in the environment of poor RNTCP covered area (tribal areas) can be suspected highly as the source of drug resistant TB. Unfavourable outcome of retreatment TB cases and poorly accessed health facility areas (tribal area) both could be consider as common attributed factors for drug resistant TB [6].

In Eastern India retreatment TB related research work among tribal population is scarce. In this background present study was conducted to assess the treatment outcome of different category retreatment cases with the aim of finding out the important predictors of unfavorable outcomes.

## 2. Methodology

This prospective cohort study was conducted in three tuberculosis units (TUs) of west Midnapore (a district of Eastern India), covering mostly the tribal populated areas. Those 3 TUs were Hijli, Digri, and Sabang. West Midnapore is a district of Eastern India where 14.87% of population are belonging to tribal family and are maintaining their tribal culture. In this rural district tuberculosis treatment services are provided from 11 TUs, 52 district microscopy centres, 119 peripheral health institutes, 25 sputum collection centres, and 945 DOT centers. We purposively selected 25% of TUs (3 TUs) where tribal inhabitants are more.

All the tribal people who were registered for Category II antituberculosis treatment between 1st quarter of 2013 (Jan to Mar) to 4th quarter of 2013 (Oct to Dec) were considered as our study cohort. They were followed up until their treatment was completed as per DOTS guideline that is December 2014. Patients, who had incomplete follow-up data in register and who were transferred out during the treatment, were excluded from the study. All the data related to exposure and outcomes were collected from tuberculosis unit's register and their follow-up data were also tracked from same secondary data source.

**Figure 1:**
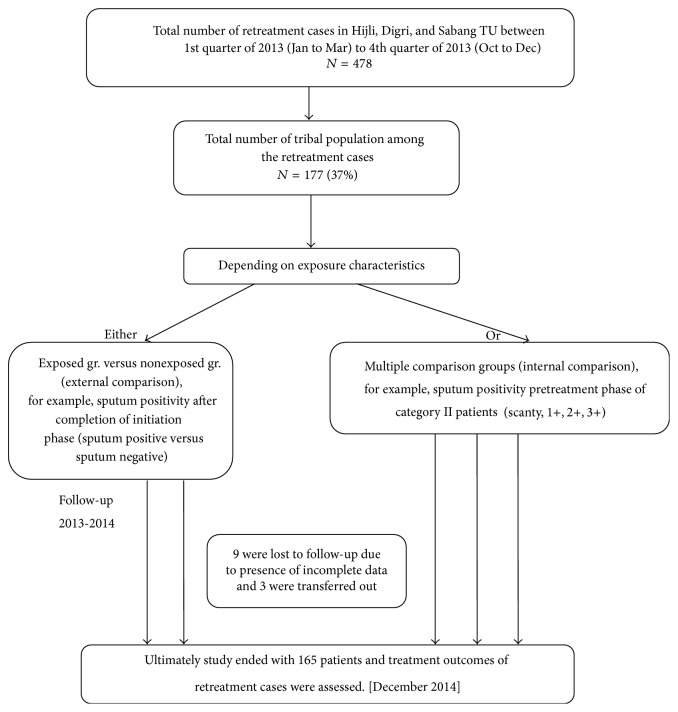
Schematic diagram showing the study design.

Complete enumeration method was adopted here. In the year of 2013, 478 retreatment cases were registered in those 3 TUs. Among them 37% (177) cases were belonging to tribal population. Tribal population were identified, by observing the surname lists under the tribal caste with clarification from the General Administration, as and when necessary. The study was started with these 177 patients but ultimately ended with 165 patients, because 9 patients were excluded due to incompletion of data and 3 were transferred out during the treatment (Figure 1).

## 3. Operational Definitions according to RNTCP Guideline [7]

### 3.1. Category of Patients Present among Retreatment Case Cohort

*Relapse*. It includes a TB patient who was declared cured or completed treatment by a physician and who reports back to the health facility and is now found to be sputum smear positive.* Treatment after default*. It includes a patient who has received treatment for TB for a month or more from any source and returns for treatment after having defaulted, that is, not taking anti-TB drugs consecutively for two months or more and found to be smear positive.* Treatment failure. *It includes any TB patient who is smear positive at 5 months or more after initiation of treatment.* Others. *It includes patient who does not fit into the any of the types mentioned above. The reasons for labeling a patient under this type must be specified in the Treatment Card and TB Register.

### 3.2. Treatment Outcome

- *Favourable Outcome*. Cured: it includes initially sputum smear positive patient who has completed treatment and had negative sputum smears on two occasions, one of which was at the end of the treatment. Treatment completed: it includes initially sputum smear positive patient who has completed treatment with negative smears at end of the intensive phase/two months in the continuation phase, but none at the end of the treatment is declared as treatment completed or initially sputum smear negative patient who has received full course of treatment and has not become smear positive at the end of the treatment.
- *Unfavourable Outcome*. Died: it includes patient who died during the course of treatment regardless of any cause. Failure: it includes any TB patient who is smear positive at five months or more after initiation of the treatment and not put on MDR-TB treatment. Defaulted: it includes a patient after treatment initiation has interrupted treatment consecutively for >2 months.

Patients were categorized according to following variables like age, gender, address, religion, type of Category II patient (relapse, failure, and treatment after default), and level of sputum positivity. These variables were considered as exposure characteristics.

Statistical analysis: data were entered in Microsoft Excel worksheet (Microsoft, Redwoods, WA, USA) and were analyzed using IBM SPSS software, version 19.0 (Statistical Package for the Social Sciences Inc., Chicago, IL, USA) and Microsoft Excel. Chi-square test was performed for bivariate analysis. Variables which were found statistically significant (*P* = 0.05) in bivariate analysis were considered for logistic regression model and adjusted odd ratio was assessed for predictor variables. Relative risk and attributed risk were also assessed for significant predictors of unfavourable outcome.

## 4. Result

### 4.1. Baseline Characteristics of the Cohort

Among 165 tribal patients, 53.9% were belonging to adolescent and young adult group, 84.2% were male, 81.8% were Hindu, and 69.7% were residing at rural community. Most of the cases of Category II patients were relapsed case (33.9%) and 29.7% of the cases were defaulter after preliminary treatment. In pretreatment period, most of the cases (31.5%) were 2+ sputum positive. After receiving initiation phase of Category II treatment 9.7% remained sputum positive. Unfavorable outcome was observed among 24.8% patients. Among them mostly 51.2% were defaulter, 22% were failure case, and 26.8% of patients died during treatment.

### 4.2. Bivariate Analysis

As all the variables (exposure characteristics) were qualitative in nature, chi-square test/Fisher exact test were applied to find out the association with treatment outcomes. The associations of unfavorable outcomes were found significantly more among adolescent and young adult patients (73.2%), treatment after default category patients (46.3%), patients who were 2+ & 3+ sputum positive at pretreatment phase (39% + 31.7% = 70.7%) and who were sputum positive after completion of initiation phase (75.6%). The chances of unfavorable outcomes were observed significantly more among minority by religion patients in comparison to favorable outcomes (34.1% versus 12.9%) (Table 1).

Among all types of unfavorable outcomes, defaulters were found as most common treatment outcome (51.2%). Defaulted treatment outcomes were observed mostly among adolescent and young adult patients (53.3%), minority by religion (71.5%), and treatment after default Category II patients (52.6%). After completion of initiation phase 50% of the sputum positive patients completed their treatment period as failure cases. About half (46.1%) of the pretreatment phase (3+) sputum positive patients died during Category II treatment phase (Table 2).

### 4.3. Logistic Regression

Factors which were found statistically significant in bivariate analysis were considered for logistic regression to measures the relationship between the categorical dependent variable (unfavorable outcome) and one or more independent variables. The logistic regression model was significant, as evident from omnibus chi-square test (*P* = 0.00). All the independent variables together can explain between 21.7% and 32.1% variance of the dependent variable (unfavorable outcome), as evident from Cox & Snell and Nagelkerke *R* square. Regression model can correctly predict 91.9% of favorable outcome and 36.6% of unfavorable outcome. Overall, the model predicts 78.2% of the outcome correctly, as shown by classification table. Ultimately in logistic regression model, religion and sputum positivity after completion of initiation phase were predicted as the significant variable of unfavorable outcome. Patients, who were minority by religion, were found 4 times more vulnerable for unfavorable outcome (Odd's Ratio = 4.1 (95% Confidence Interval = 1.6–10.8), *P* = 0.004). Unfavorable outcome was found 7 times more common among retreatment TB cases who remain sputum positive after completion of initiation phase of Category II treatment (Odd's Ratio = 6.9 (95% Confidence Interval = 1.9–24.7), *P* = 0.003) (Table 3).

### 4.4. Relative Risk and Attributed Risk

Relative risk and attributed risk were calculated for the significant predictors. Relative risk showed that patients, who were sputum positive after completion of initiation phase, had 2.98 times more risk than others for the development of unfavorable outcome. In case of minority by religion that risk was 2.35 times more than Hindu tribal. Attributed risk indicated that 66.4% of unfavorable outcome occurred due to failure of sputum conversion after initiation phase of treatment. Religion of retreatment cases attributed 57.4% cases of unfavorable outcome when they were belonging to minority by religion (Table 4).

## 5. Discussion

This prospective cohort study was conducted during 2013 to 2014 and it was started with the tribal retreatment case cohort who were registered as Category II tuberculosis cases between 1st quarter of 2013 (Jan to Mar) and 4th quarter of 2013 (Oct to Dec) in three tuberculosis units (TUs) of west Midnapore (a district of Eastern India) and ended on December 2014. The main reasons behind the unfavorable outcomes of retreatment cases are irregular DOTS therapy, poor coverage of RNTCP programme, quality of drugs, and health care. Tribal inhabitants usually lived in hard to reach areas where RNTCP coverage is poor and treatment outcome of Category II cases is also unsatisfactory [8]. So the present study was conducted on this special group of people to assess the treatment outcome of different category retreatment cases with the aim of finding out the important predictors of unfavorable outcomes.

In the present study we have found 75.2% favourable outcome that is similar to the treatment success rate (71%) of India in retreatment TB cases [9]. Among the unfavourable outcomes more were defaulter (51.2%) that also collaborate with the Vijay et al. (72.8%) and Vasudevan et al. (37.6%) study, but in our study, rate of defaulters was quite less than Vijay et al. study and quite higher than Vasudevan et al. study [10, 11]. Though the rate of defaulters was varied widely within India but in all studies it was counted as most common unfavourable outcome. More numbers of defaulters indicate the failure of programme implementation like poor coverage and lack of tracking activity of defaulters under the RNTCP. In the present study, unfavorable outcomes were found significantly more among adolescent and young adult patients (73.2%), minority by religion (26.8%), and treatment after default category patients (46.3%) and patients who were 2+ & 3+ sputum positive at pretreatment phase and who were sputum positive after completion of initiation phase. Vijay et al. study found treatment after default category patients as potential defaulters/unfavourable outcome that is also supported by our study findings. Both Vijay et al. and Dandekar et al. study found gender as significant predictors of overall unfavourable treatment outcome, but we did not find any significant relationship between gender and unfavourable treatment outcome [10, 12]. We observed unfavourable outcome commonly among high grade sputum positive patients that is also collaborative with the Mukherjee et al. study observations. They also found unfavourable outcome most likely among treatment failure subgroup but we observed it more among treatment after default category patients (46.3%) [13].

Ultimately logistic regression model found that patients, who were minority by religion, were 4 times more vulnerable for unfavorable outcome (Odd's Ratio = 4.1 (95% Confidence Interval = 1.6–10.8), *P* = 0.004) and unfavorable outcomes were also found to be 7 times more common among retreatment TB cases who remain sputum positive after completion of initiation phase of Category II treatment (Odd's Ratio = 6.9 (95% Confidence Interval = 1.9–24.7), *P* = 0.003). This regression model can predict only 36.6% of unfavorable outcome correctly. Programmatic variables were not assessed in our study that can explain the low predictability of regression model. In spite of poor predicted ability of the regression model, it predicted two most important predictors of unfavourable outcome that should be intervened to improve the treatment outcome of retreatment TB cases.

In our study patients who were minority by religion were more susceptible to unfavourable outcomes, but Dandekar found no significant relationship between them and no such observation was found in other studies also. It is more hard for patients who are minority among tribals to reach group due to their cultural and communication barrier. That might explain their unfavorable outcomes. Sputum conversion rate in our study was 90.3% and that is quite higher than Vasudevan et al. study's observation (76.9%). Patients who remain sputum positive after completion of initiation phase of Category II treatment were found more susceptible to unfavourable outcome (OR = 6.9) that is also suggested by Dooley et al. study (OR = 7.14) in Morocco [14]. In our study, although there was no such scope to measure drug sensitivity and resistance, it could be assume that more unfavourable outcome among unsuccessful sputum converted cases probably due to development of drug resistance. It might be single drug or multidrug resistance. If the MDR-TB diagnostics resources facility scaled up everywhere then the problem could be figure out more elaborately and results would be more evidence based.

Though the present study was not any comparison between tribal and nontribal population, its outcome was found more or less same as overall treatment outcome. Although poor living conditions, malnutrition, erroneous health assumptions and beliefs concerning TB, lack of resources, and treatment by traditional healers increase the burden of TB among the tribal/indigenous population [15–17], the treatment outcome is not significantly associated with ethnicity that was also observed in a previous study conducted by Chakrabarti et al. in West Bengal [18]. It indirectly indicates that programme failure related variables like lack of access to proper treatment, traditional and cultural barriers, lack of tracking of defaulters, and so forth, which were not addressed in previous study and were more important predictors than sociodemographic characteristics and it was felt by Chakrabarti et al. also [18]. That means unfavourable outcome among tribal population can be avoided, because it less depends on nonmodifiable factors like ethnicity.

## 6. Conclusion

This study was conducted among tribal population in assumption that unfavourable outcome will be found more prevalent among them. But the study results collaborated with the overall treatment outcome of India. In the end of the study patients who were minority by religion and who remained sputum positive after completion of initiation phase of treatment were found more susceptible to unfavourable outcome. Programmatic approach should be specified to address the minority by religion population by breaking the communication barrier. Tracking of defaulters and early diagnosis of drug resistance cases by scaling up diagnostic facilities can reduce the load of sputum positive cases after completion of initiation phase treatment as well as unfavourable outcome also. In future studies if the programme related variables could be addressed, more important predictors of retreatment cases outcome will be revealed.

## Figures and Tables

**Table 1:** Bivariate analysis between patient's profile and treatment outcome. *N* = 165.

Patient's profile	Favourable outcome *N* (%)	Unfavourable outcome *N* (%)	Chi-square test
Age			
Adolescent & young			*χ* ^2^ = 10.066 df = 2 *P* = 0.007^*∗*^
Adult (11–40)	59 (47.6)	30 (73.2)	
Middle age (41–60)	59 (47.6)	8 (19.5)	
Geriatric > 60	6 (4.8)	3 (7.3)	

Gender			
Male	104 (83.9)	35 (85.4)	*χ* ^2^ = 0.052 df = 1 *P* = 0.82
Female	20 (16.1)	6 (14.6)	

Address			
Rural	86 (69.4)	29 (70.7)	*χ* ^2^ = 0.028 df = 1 *P* = 0.868
Urban	38 (30.6)	12 (29.3)	

Religion			
Hindu	108 (87.1)	27 (65.9)	*χ* ^2^ = 9.838 df = 2 *P* = 0.007^*∗*^
Minority by religion	16 (12.9)	14 (34.1)	

Type of category II patients			
Treatment after default	30 (24.2)	19 (46.3)	*χ* ^2^ = 15.591 df = 4 *P* = 0.004^*∗*^
Failure	6 (4.8)	5 (12.2)	
Relapse	43 (34.7)	13 (31.7)	
Transferred in	35 (28.2)	2 (4.9)	
Others	10 (8.1)	2 (4.9)	

Sputum positivity pretreatment phase of category II			
Scanty	47 (37.9)	3 (7.3)	*χ* ^2^ = 18.338 df = 3 *P* = 0.000^*∗*^
1+	27 (21.8)	9 (22)	
2+	36 (29)	16 (39)	
3+	14 (11.3)	13 (31.7)	

Sputum positivity after completion of initiation phase			
Negative	118 (95.2)	31 (75.6)	Fisher exact test *P* value = 0.001^*∗*^
Positive	6 (4.8)	10 (24.4)	

^*∗*^ *P* value is significant (<0.05).

**Table 2:** Distribution of tribal patients according to the patient's profile and treatment outcome. *N* = 165.

	Favourable outcome		Total	Unfavourable outcome			Total
	Cured	Treatment completed		Failure	Defaulted	Died	
Age							
Adolescent & young adult (11–40)	41 (69.49)	18 (30.51)	59 (100)	6 (20)	16 (53.3)	8 (26.7)	30 (100)
Middle age (41–60)	38 (64.4)	21 (35.6)	59 (100)	2 (25)	3 (37.5)	3 (37.5)	8 (100)
Geriatric > 60	3 (50)	3 (50)	6 (100)	1 (33.3)	2 (66.7)	0 (0)	3 (100)

Gender							
Male	71 (68.3)	33 (31.7)	104 (100)	8 (22.9)	17 (48.5)	10 (28.6)	35 (100)
Female	11 (55)	9 (45)	20 (100)	1 (16.7)	4 (66.6)	1 (16.7)	6 (100)

Address							
Rural	55 (64)	31 (36)	86 (100)	7 (24.1)	16 (55.2)	6 (20.7)	29 (100)
Urban	27 (71.1)	11 (28.9)	38 (100)	2 (16.6)	5 (41.7)	5 (41.7)	12 (100)

Religion							
Hindu	68 (63)	40 (37)	108 (100)	8 (29.6)	11 (40.6)	8 (29.6)	27 (100)
Minority by religion	14 (87.5)	2 (12.5)	16 (100)	1 (7.1)	10 (71.5)	3 (21.4)	14 (100)

Types of category II patient							
Treatment after default	29 (96.7)	1 (3.3)	30 (100)	3 (15.8)	10 (52.6)	6 (31.6)	19 (100)
Failure	6 (100)	0 (0)	6 (100)	3 (60)	2 (40)	0 (0)	5 (100)
Relapse	36 (83.7)	7 (16.3)	43 (100)	2 (15.4)	7 (53.8)	4 (30.8)	13 (100)
Others	8 (80)	2 (20)	10 (100)	1 (50)	1 (50)	0 (0)	2 (100)
Transferred in	3 (8.6)	32 (91.4)	35 (100)	0 (0)	1 (50)	1 (50)	2 (100)

Sputum positivity pretreatment phase of category II patients							
Scanty	10 (21.3)	37 (78.7)	47 (100)	1 (33.3)	1 (33.3)	1 (33.3)	3 (100)
1+	25 (92.6)	2 (7.4)	27 (100)	1 (11.1)	6 (66.7)	2 (22.2)	9 (100)
2+	33 (91.7)	3 (8.3)	36 (100)	4 (25)	10 (62.5)	2 (12.5)	16 (100)
3+	14 (100)	0 (0)	14 (100)	3 (23.1)	4 (30.8)	6 (46.1)	13 (100)

Sputum positivity after completion of initiation phase							
Negative	77 (65.3)	41 (34.7)	118 (100)	4 (12.9)	19 (61.3)	8 (25.8)	31 (100)
Positive	5 (83.3)	1 (16.7)	6 (100)	5 (50)	2 (20)	3 (30)	10 (100)

**Table:** (a) Omnibus tests of model coefficients

		Chi-square	df	Sig.
Step 1	Step	40.272	8	.000
	Block	40.272	8	.000
	Model	40.272	8	.000

**Table:** (b) Model summary

Step	−2 log likelihood	Cox & Snell *R* square	Nagelkerke *R* square
1	144.747^a^	.217	.321

^a^Estimation terminated at iteration number 6 because parameter estimates changed by less than .001.

**Table:** (c) Classification table^a^

	Observed		Predicted		
			Outcome		Percentage correct
			.00	1.00	
Step 1	Outcome	Favorable (0.00)	114	10	91.9
		Unfavorable (1.00)	26	15	36.6
	Overall percentage				78.2

^a^The cut value is .500.

**Table:** (d) Variables in the equation

		*B*	SE	Wald	df	Sig.	Exp(*B*)	95% CI for EXP(*B*)	
								Lower	Upper
Step 1^a^	(1) Age	−.014	.016	.723	1	.395	.986	.955	1.018
	(2) Religion	1.415	.493	8.226	1	.004	4.115	1.565	10.821
	(3) Type of category II patients			6.099	4	.192			
	Treatment after default (1)	1.416	.896	2.494	1	.114	4.120	.711	23.876
	Failure (2)	.985	1.080	.831	1	.362	2.678	.322	22.241
	Relapse (3)	.671	.898	.558	1	.455	1.955	.336	11.367
	Transferred in & others (4)	−.529	1.226	.186	1	.666	.589	.053	6.515
	(4) Sputum positivity pretreatment phase of category II patients	.358	.250	2.042	1	.153	1.430	.876	2.336
	(5) Sputum positivity after completion of initiation phase	1.937	.649	8.920	1	.003	6.940	1.946	24.746
	Constant	−4.402	1.359	10.491	1	.001	.012		

^a^Variable(s) entered on step 1: age, religion, type of category II patients, sputum positivity pretreatment phase of category II patients, and sputum positivity after completion of initiation phase.

#Scoring:

Religion: Hindu = 0, minority by religion = 1; type of category II patients: treatment after default = 0, failure = 1, relapse = 2, and transferred in & others = 3; sputum positivity pretreatment phase of category II patients: (scanty & 1+) = 0, (2+ & 3+) = 1; sputum positivity after completion of initiation phase: negative = 0, positive = 1.

**Table 4:** Relative risk and attributed risk for significant predictors of unfavorable outcome.

Predictors of unfavorable outcome	Relative risk	Attributed risk (%)
Religion	2.35	57.4
Sputum positivity after completion of initiation phase	2.98	66.4

## Additional Points

Institutions in which the work was conducted are Tuberculosis Unit of Midnapore District, West Bengal, and Department of Community Medicine, Midnapore Medical College and Hospital. The study is self-funded.
